# The Targeted Metabolomic Signatures of Phytohormones in Leaves of Mulberry (*Morus alba* L.) Are Crucial for Regrowth and Specifically Modulated by the Differential Stubble Lengths

**DOI:** 10.3390/plants14071126

**Published:** 2025-04-05

**Authors:** Haonan Li, Michael Ackah, Frank Kwarteng Amoako, Aaron Tettey Asare, Jianbin Li, Zhenjiang Wang, Qiang Lin, Changyu Qiu, Mengdi Zhao, Weiguo Zhao

**Affiliations:** 1 Jiangsu Key Laboratory of Sericulture Biology and Biotechnology, School of Biotechnology, Jiangsu University of Science and Technology, Zhenjiang 212100, China; 18501555989@163.com (H.L.); 13204812646@163.com (J.L.); 2 Key Laboratory of Silkworm and Mulberry Genetic Improvement, The Sericultural Research Institute, Chinese Academy of Agricultural Sciences, Ministry of Agriculture and Rural Affairs, Zhenjiang 212100, China; 3 Department of Molecular Biology and Biotechnology, School of Biological Sciences, College of Agriculture and Natural Sciences, University of Cape Coast, Cape Coast 00233, Ghana; kwamekwarteng242@gmail.com (F.K.A.); aasare@ucc.edu.gh (A.T.A.); 4 Institute of Plant Nutrition and Soil Science, Kiel University, Hermann-Rodewald-Straße 2, 24118 Kiel, Germany; 5 Sericulture & Agri-Food Research Institute, Guangdong Academy of Agricultural Sciences, Guangzhou 510610, China; wzhjiang@126.com; 6 Sericulture Technology Promotion Station, Nanning 530007, China; gxlq67@163.com (Q.L.); changyuqiu2008@163.com (C.Q.); 7 Department of Materials Science and Engineering, Suzhou University of Science and Technology, Suzhou 215011, China

**Keywords:** mulberry, *Morus alba* L., stubble, targeted metabolomics, phytohormones, abscisic acid, gibberellin

## Abstract

Vegetative propagation of mulberry (*Morus alba* L.) via sapling methods, due to the ability to exponentially multiply lateral buds on stem cuttings to enhance rapid shoot formation, is crucial for sericulture industries. The sprouting of mulberry using stubbles is an emerging method for rapid and mass production of mulberry leaves, but the growth mechanisms associated with its use remain obscure. This study is the first to report how the differential stubble lengths from mulberry plants alter and modulate phytohormones and the associated mechanisms. This study seeks to evaluate the growth mechanisms by elucidating the phytohormone signature modulation in response to differential stubble lengths of 0 cm, 5 cm, 10 cm, 20 cm, and a control via targeted metabolomics analysis in mulberry leaves. The results consistently show that the use of differential stubble lengths of mulberry promoted growth, the number of buds, aboveground biomass, and branch and leaf weights by improving the net photosynthesis, transpiration rate, stomatal conductance, and intercellular CO_2_ relative to the control. The differential stubble lengths not only caused contrasting responses in the contents of plant hormones, including salicylic acid (SA), abscisic acid (ABA), indole-3-acetic acid (IAA), jasmonic acid (JA), and gibberellin (GA), but also modulated higher elemental contents relative to the control. The results further reveal significant and positive correlations between the phytohormones and all growth, biomass, and photosynthetic parameters, highlighting the role of phytohormones in the sprouting and rejuvenation of mulberry stubbles. Meanwhile, the targeted metabolomics analysis identified a total of 11 differentially accumulated phytohormones in response to the differential stubble lengths, which were significantly implicated and enriched in three major pathways, including the biosynthesis of plant hormones (ko01070), metabolic pathways (ko01100), and the plant hormone signal transduction pathway (ko04575). The use of stubbles for rapid leaf production in mulberry plants is of great importance to improve early sprouting and cutting survival, as well as shortening growth and rooting time, and is highly recommended for the sericulture industries.

## 1. Introduction

Mulberry plants, belonging to the genus Morus, are an intriguing group of deciduous wind-pollinated perennial trees and shrubs distinguished for their rapid growth, vibrant fruits and leaves for feeding silkworms in the sericulture industries around the globe [1,2,3,4]. Cultivation of mulberry ranges from native to warm temperate and subtropical regions, predominantly in Asia, some parts of Africa, and the Americas. Mulberry plants are known to thrive in a variety of environments [5,6,7,8]. Many cultivars and species have been identified, but the most cultivated species include the white mulberry (*Morus alba*), black mulberry (*Morus nigra*), and red mulberry (*Morus rubra*) [9]. Although mulberry is a perennial tree plant, it is propagated via both sexual and asexual modes of propagation. As a result of its heterozygous nature and long juvenile period, with seed propagation estimated to bear fruits in a decade, the asexual or vegetative means of propagation has been the most used method in its cultivation [10]. It is not only complaisant to propagation via the seed sowing, the generation of seedlings, but also responsive to cutting, grafting, budding, and layering methods in a process called saplings [11]. It has recently been found that generating mulberry saplings through stem cutting is fascinatingly the most principal and conspicuous mode utilized across all the mulberry-cultivating countries [11]. This is due to its ability to profusely produce succulent leaves for feeding silkworms. Depending on the cultivar, a well-developed and healthy shoot with active buds from 6-to-8-month-old stem cutting is reportedly suitable for propagation [11]. Early sprouting of these cuttings depends on the size, length and the number of the buds on the cuttings. For instance, cuttings with lengths ranging from an estimated 15 to 20 cm, with about 3–4 active buds, and 22–25 cm long, with 5–6 healthy buds, are recommended for generating and sprouting mulberry under conducive environmental conditions [11]. Indeed, the rooting capabilities of mulberry via cuttings depends not only on the inherent factors such as genotype (regenerative or rejuvenation ability), but other determinants, including environmental factors (temperature, humidity etc.), physiological factors (development of root primordial, amount of nutrient stored), management and intercultural operations (fertilization, irrigation, weeding, etc.), age and quality of the stem cuttings, and exogenous hormone treatments [11,12]. Apart from the above factors, the rooting abilities of cuttings largely depend on whether it is a temperate, tropical or sub-tropical genotype. Recent evidence suggests that the tropical mulberry cultivar cuttings have higher rooting propensity relative to the temperate mulberry cultivars [11,13]. However, the cuttings of the temperate varieties of mulberry with low rooting abilities are not only enhanced with diverse rooting or growth-regulating hormones, including indole acetic acid, indole butyric acid, and naphthalene acetic acid, but can also be improved by grafting a poor rooter as scion on a good rooter variety as stock [11,13].

Vegetative propagation via stem cutting is regarded as a post-operation root regeneration and rejuvenation processes in plant growth and development [14]. Cuttings made from parent plants immediately lose nutrients, substrates and water in the process [12,15], leading to inhibition of adventitious root formation that ensures rejuvenation and regeneration of new roots from buds. To counteract plasticity such as oxidative stress and redox imbalances in post-cuttings, plants induce endogenous hormones to trigger the formation of adventitious roots. Additionally, the treatment of cuttings with exogenous hormones, including abscisic acid (ABA), gibberellins (GAs), ethylene (ETH), auxin, indole-3-acetic acid (IAA), cytokinins (CKs), brassinosteroids (BRs), and melatonin (MLT), promotes root formation in the plant [12,16]. Adventitious root formation in cuttings is strictly modulated by a large set of exogenous and endogenous hormones, which coordinate many physiological and biochemical processes. Early studies have opined that exogenous hormone treatment accelerates cell division, improves biosynthesis of intrinsic hormones, salicylic acid, accumulation of carbohydrate, and consequently stimulates adventitious root formation [12,16,17]. Similar reports have suggested that auxin is the master regulator coordinating and controlling adventitious root formation, and plays a decisive role in cell fate, and activates signaling networks in plants [18]. For example, multiple experimental substantiations have reported that the treatments of auxin stimulated adventitious root formation by triggering auxin-promoted cell wall loosening and stretching in plants such as black walnut, black locust and *Populus tremula* [19,20,21,22]. Stem cuttings of *M. hupehensis* subjected to IAA, naphthalene acetic acid (NAA), or green growth regulator (GGR) not only showed apparent improvement in adventitious root at stages, including the root pre-emergence stage, the early stage of root formation, the massive root formation stage, and the later stage of root formation, but also caused a concomitant reduction in rooting time by 25–47.4% and promoted the rooting efficiencies of cuttings by 0.9–1.3 times, relative to the control [12]. Additionally, exogenous IAA significantly promoted plant height, stem diameter, leaf area and number as well as the contents of endogenous IAA and GA3, but ABA content was significantly decreased in Syringa plants [23]. Indeed, the possible synergistic and antagonistic interactions among different phytohormones cannot be over-ruled. Multiple research attestations have highlighted some of these interactions. For instance, ETH was found to have positively regulated adventitious root formation via adjusting auxin transport and distribution in tomato [24]. Meanwhile, CKs interacted with ETH and auxin pathways to repress adventitious root development in poplar [25]. Furthermore, high-throughput metabolomics analyses have identified diverse differentially accumulated phytohormone metabolites. For example, a study optimizing the cultivation technique to shorten the cultivation cycle of *Pueraria montana* plants (var. Gange No. 5″) from two years to one year identified 42 differentially accumulated hormone metabolites, with hormones, including auxin, CKs, jasmonic acids (JAs), salicylic acid (SA), MLT, ETH, ABA, etc., accumulating maximum contents at the pre- and final-expansion periods [26]. Such hormones were suggested to play a heightened role in stimulating the initiation of tuberous root expansion.

Based on these premises and antecedents, it is quite plausible to highlight that ensuring homeostasis by maintaining the endogenous hormone balance in rooting during vegetative propagation process is crucial. The possible question or hypothesis is, do the differences in lengths/heights of a stem cutting stimulate and induce biosynthesis of endogenous hormones, which consequently triggers the formation of adventitious roots and general growth of plants? Evidence has suggested and confirmed that the treatment of stem cuttings with phytohormones not only stimulates adventitious root formation, regeneration and rejuvenation of plants but also improves the general growth, as elucidated earlier [23,26]. However, the counter argument is that the differences in lengths of stem cuttings stimulate and modulate the accumulation of endogenous phytohormones in plants remain obscure. Hence, investigating how varying lengths of stubbles in plants induce endogenous phytohormones that promote and initiate adventitious root formation during vegetative propagation is of great significance and will likely reveal striking evidence in current research. To the best of our knowledge, this study happens to be the first study elucidating the mechanisms and how the ripple effects of differential lengths of stubbles stimulate and accumulate endogenous phytohormones in mulberry plants via morpho-physiological and targeted metabolomics analyses.

We aim to compare and elucidate the mechanisms and the repercussions of the differential length of stubbles on regrowth, physiological and hormone accumulation of mulberry parameters, as well as the signatures in phytohormone metabolites using targeted metabolomics. If successful, the mechanisms and alterations in the biosynthesis of plant hormones and plant hormone signal transduction pathways that manifest as a result of the differential stubble lengths of mulberry would be the prime focus of this study. Shedding light on the variations in stubbles of mulberry plants is of great importance to improve the cutting survival and shortening of rooting time for the rapid production of leaves for the sericulture industries.

## 2. Materials and Methods

### 2.1. Experimental Materials and Design

Mulberry (*M. alba* L.) was collected from the National Mulberry GenBank at Jiangsu University of Science and Technology, Zhenjiang, Jiangsu, China. Field experimental design was used and adopted a single-factor regression design. The experiment combined randomized arrangement and Latin square arrangement for the field setup. The stubble area underwent complete stumping, with four stubble lengths (0 cm, 5 cm, 10 cm, and 20 cm) as treatments and unstumped plants as the control (CK). The plants used for the stubble were 2 years old on loamy soil field. The soil was supplemented with NPK (14:16:15) compound fertilizer at a rate of 122.5 g per meter square (applied at one time in May 2024). The experimental set-up consisted of 5 treatments, with each treatment containing 5 plots as replicates, totaling 25 plots. Each treatment contained 10 stubbles, resulting in a total of 200 stubbles and 50 normal plants making 250. The experiment was performed on 18 June 2024.

### 2.2. Field Investigation and Sampling

**Figure 1:**
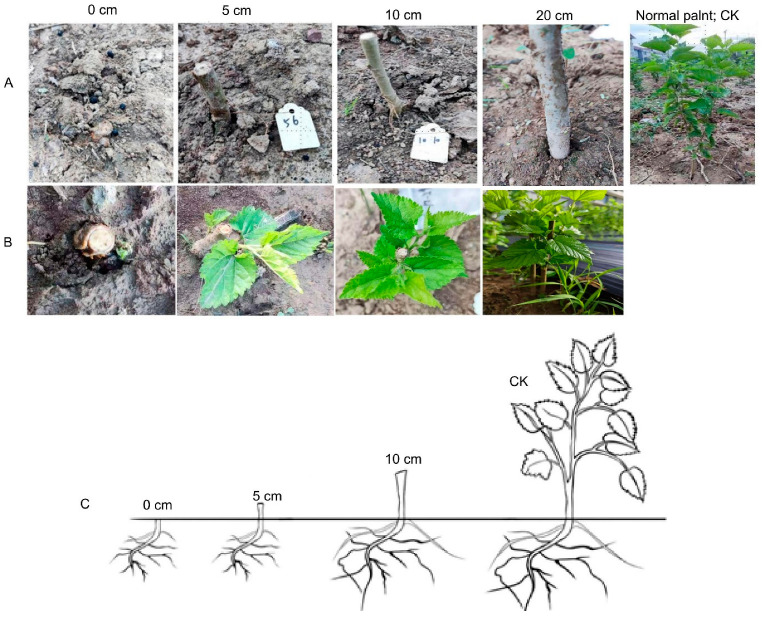
Various stubble lengths illustrating the treatments and the plant regrowth. (**A**) Stubble lengths and the normal plant. (**B**) Sprouting and regrowth of stubbles. (**C**) Stubble lengths illustration.

After the experimental setup, follow-up investigations were conducted to monitor the sprouting and re-growth conditions of the stumped plants (stubbles), as shown in Figure 1. The investigation included the checking of the number of sprouts by counting and tagging, sprout growth by measuring the sprouts’ growth with ruler; new shoot growth of CK plants and the stubbles were also measured. The investigation period spanned from June 2024 to October 2024, with growth indicators measured every week. Leaf sampling was performed by selecting three standard plants from each stubble group. First, leaf samples were collected 15 days (used for metabolomics and biochemical analysis) after sprouting of the stubble. Again, leaf samples were collected at 90 days of stubble growth (used for other analysis including mineral analysis). For the stubble plants, young leaves from the sprouting shoots were collected. For normal plants, young leaves from the middle part of the canopy were collected. Each sample (approximately 0.5 g) was placed into a cryotube. Three tubes were collected for each replicate at each sampling time. To ensure the samples were dry and uncontaminated, latex gloves and masks were worn during sampling. After collection, the leaf samples were quickly transferred into cryotubes and placed in a liquid nitrogen tank. Upon returning to the laboratory, the samples were stored in an ultra-low temperature freezer at −80 °C for preservation.

### 2.3. Biomass Measurement

After 90 days of stubbles growth, the biomass of plants was measured. To measure the biomass, 10 stubble plants and 10 normal plants were randomly selected for biomass measurement. The types of biomass measured include aboveground biomass, fresh branch weight, fresh leaf weight and dry leaf weight. A precision electronic balance (BSA224S, Sartorius, Beijing, China) with accuracy 0.001 g was used for the measurement. Fresh branch weight was the weight of branches after leaf removal. Fresh leaf weight was calculated by subtracting the branch weight from the total fresh weight. Dry leaf weight was determined by placing leaves in separate beakers and were subjected to drying process using an electric blast drying oven (DHG-9140A, JingHong, Shanghai, China) set at 65 °C for 48 h until a constant weight was achieved [5].

### 2.4. Photosynthetic Parameters and Chlorophyl Measurement

Photosynthetic parameters were measured using the PPSYSTEMS CIRAS-3 Portable Photosynthesis System (PP Systems, Amesbury, MA, USA) analyzer from 9:00 a.m. to 11:00 a.m. Leaves from the 3rd to 5th leaf positions facing the sun, and with uniform growth, were selected for measurement. Parameters measured included net photosynthetic rate (Pn), transpiration rate (Tr), stomatal conductance (Gs), intercellular CO_2_ concentration (Ci), and water use efficiency (WUE). Leaf temperature was at 31.1 ± 2 °C and light intensity was at 1000 μmol/m^−2^ s^−1^. The relative humidity was around 35%. Leaf chamber was 18 cm × 25 cm square window, using an open gas exchange system. Flow rate was set to 500 μmol·s^−1^. A buffer bottle was used to stabilize the atmospheric CO_2_ concentration (CO_2_) between 380 and 420 μmol·mol^−1^. Three plants with consistent growth were randomly selected from each stubble group and leaves from the same position were measured [5], with three replicates per leaf and two technical replicates. Subsequently, analysis of variance and statistical significance (Tukey’s HSD, *p* < 0.05) was determined using R software v4.2, and the graphs were visualized using Hiplot Pro (https://hiplot.com.cn/ accessed on 21 February 2025), a comprehensive web service for biomedical data analysis and visualization. Total Chlorophyl (Chl) content was measured using SPAD-502 chlorophyl meter (Konika Minolta, Tokyo, Japan).

### 2.5. Minerals and Crude Protein Determination

Mineral content in *M. alba* leaves was determined using an inductively coupled plasma-atomic emission spectroscopy/mass spectrometry (ICP-AES/OES/MS) apparatus (PerkinElmer, Waltham, MA, USA). This analysis was performed according to the standards of the People’s Republic of China National Food Safety Standard for the analysis of multiple elements in food (GB 5009.268–2016) [27]. Using mixed sampling method, the leaves of M. alba harvested 90 days after stubble sprout and the CK were used for both minerals and crude protein analysis. Sample preparation followed the study by Li et al. (2024) [5]. Samples were washed with deionized water and then dried with absorbent paper and then oven-dried to constant weight at 65 °C. The dried leaves were then ground with mortar to a fine powder. The leaf powder (0.2 g) was weighed in the PTFE (polyteflon) digestion tube and soaked overnight with 5 mL nitric acid. The digestion tubes were tightened and placed into microwave at 80 °C for 2 h; the temperature increased from 120 °C to 160 °C over 2 h. The digested product was transferred into a 25 mL volumetric flask and diluted with H_2_O (100 mL) and stored for content analysis [5,8]. A blank, without sample was prepared. The element contents were measured separately with ICP-AES/OES/MS. Each group comprised three biological replicates, with each replicate having three leaves. The element concentrations in the samples were calculated based on the calibration curves and the sample’s emission intensity.

The crude protein content was determined according to the Agricultural Industry Standard of the People’s Republic of China (NY/T 3-1982) [28]. Briefly, dry leaf samples (three replicates each) from the stubble groups and the CK were used for the crude protein determination. The leaf samples (0.20 g) were digested with concentrated sulfuric acid in the presence of a catalyst (selenium or copper sulfate) to convert the nitrogen in the sample into ammonium sulfate. The digested sample was then distilled with sodium hydroxide to release ammonia gas, and the ammonia was captured in a boric acid solution. The ammonia-boric acid solution was titrated with a standard acid (hydrochloric acid or sulfuric acid) to determine the nitrogen content. The nitrogen content was then converted to crude protein using a conversion factor (6.25 for most plant materials) and the crude protein was calculated as crude protein (%) = nitrogen content (%) × 6.25. Three biological replicates per leaf and two technical replicates were used.

### 2.6. Biochemical Measurements of Hormones and Correlation Analysis

To determine the contents of hormones biochemically, hormones such as salicylic acid (SA), abscisic acid (ABA), indole-3-acetic acid (IAA), jasmonic acid (JA), and gibberellin (GA) were evaluated in leaves of *M. alba* stubbles (0 cm, 5 cm, 10 cm) from plants harvested after 15 days of stubble sprouting and the normal plant (CK). All the parameters were measured with detection Kit provided by Suzhou Keming Biotechnology Co., Ltd., Suzhou, China, following the manufacturer’s instructions. Three biological replicates were used in each indicator. Data were processed and, subsequently, analysis of variance and statistical significance (Tukey’s HSD, *p* < 0.05) were determined using R software v4.2. Figures were plotted in Hiplot Pro (https://hiplot.com.cn). Pearson correlation heatmap analysis involving the hormones, elements, growth and photosynthetic parameters was performed in Corrplot tools in Hiplot Pro (https://hiplot.com.cn/). Correlation coefficient = 1 or −1 was considered positive or negative correlation, respectively, at *p* < 0.05.

### 2.7. Sample Preparation for Metabolites Extraction

All chemicals including methanol (Sigma-Aldrich, St. Louis, MO, USA), acetonitrile (Sigma-Aldrich), formic acid (Aladdin, Waukesha, WI, USA), standard substances (YuanYe, Aladdin, Sigma-Aldrich) used in this study were of high-performance liquid chromatography (HPLC) or analytical grade. M. alba leaves obtained from the various stubbles and the normal plant were used for the metabolite’s extraction. In total, 12 samples comprising 4 groups and 3 replicates each were used for the extraction and analysis. Leaf samples were thawed at 4 °C in a refrigerator, and the same group of samples were mixed well. An appropriate amount of sample (0.1 g) was placed in a 10 mL centrifuge tube, and 5 mL of extraction solution (methanol: water: formic acid; 15:4:1 with 0.5% BHT) was added. The mixture was vortexed for 1 min, ultrasonicated for 30 min, and allowed to stand at −40 °C for 60 min, then centrifuged at 12,000 rpm for 10 min. The supernatant was then removed for solid-phase extraction. In the solid-phase extraction, an eluent (activation: 3 mL water, 3 mL methanol; adsorption: the supernatant into the SPE solid phase extraction column (flow rate ≤ 1 mL/min); rinsing: 3 mL water, 10% methanol water; elution: 1 mL methanol) was performed. The above eluent was concentrated to dryness in a concentrator, re-dissolved in 0.60 mL of 80% methanol water, vortexed and mixed for 1 min, centrifuged at 12,000 rpm for 10 min, and then the supernatant was taken into the machine for LC-MS/MS analysis.

### 2.8. LC-MS/MS Analysis

The liquid chromatography system was a Waters Acquity UPLC, coupled with a mass spectrometer from AB SCIEX 5500 QQQ-MS (SCIEX, Framingham, MA, USA). The chromatographic columns employed were Acquity UPLC BEH C18 (1.7 µm, 2.1 mm × 100 mm) and Acquity UPLC HSS T3 (1.8 µm, 2.1 mm × 100 mm). The chromatographic separation conditions included a column temperature of 35 °C and a flow rate of 0.30 mL/min. The mobile phase composition consisted of component A, which was water containing 10 mM ammonium formate, and component B, which was methanol. The total runtime was 8 min, with an injection volume of 6 µL. The mass spectrometric conditions were as follows: ion source, ESI; curtain gas, 35 arb; collision gas, 7 arb; ion spray voltage, 4500 V; ion source temperature, 450 °C; ion source gases, 55 arb each for ion source gas1 and ion source gas2. The MRM acquisition parameters were established based on the aforementioned chromatographic and mass spectrometric conditions, and standard solution preparations were injected into the sample vials for analysis.

### 2.9. Data Processing and Quality Control Analysis of Metabolites

Integration was carried out using MultiQuant software (v3.0.3), and content calculation was performed according to the internal standard one-point method. The internal standard method was to add a certain weight of pure substance as an internal standard to a certain amount of the analyzed sample mixture and calculate the content of the measured component according to the mass ratio of the test sample and the internal standard, the ratio of its corresponding chromatographic peak area and the relative correction factor. The internal standard method was calculated as shown below:

$$f=\frac{\frac{A_{s}}{m_{s}}}{\frac{A_{r}}{m_{r}}}$$

From the formula, *As* and *Ar* are the peak areas or peak heights of the internal standard and the control, respectively, and *ms* and *mr* are the amounts of the internal standard and the control added, respectively. The solution of the component to be tested containing the internal standard was taken into the sample, and recorded the chromatogram, and then calculated the content (*mi*) according to the peak response value of the solution of the component to be tested containing the internal standard:

$$m_{i}=f\times \frac{A_{i}}{\frac{A_{s}}{m_{s}}}$$

where *Ai* and *As* are the peak areas or peak heights of the metabolite to be measured and internal standard, respectively, and *ms* is the amount of internal standard added. The MultiQuant software (v3.0.3) was utilized for integration and the standard curve was utilized for content calculation. Data quality control (QC) was determined mainly by the RSD value (relative standard deviation) of each targeted metabolite in the QC samples. In general, the RSD value of <10% was deemed a good data quality.

### 2.10. Multivariate Statistical Analysis

For a preliminary visualization of differences between different groups of samples, the unsupervised dimensionality reduction method principal component analysis (PCA) was applied in all samples using R package models (v2.16. 2) (http://www.r-project.org/) [29]. Again, partial least squares discriminant analysis (PLS-DA) is a supervised dimensionality reduction method in which class memberships are coded in matrix form into Y to better distinguish the metabolomics profile of two groups by screening variables correlated to class memberships [30]. PLS-DA was applied in comparison groups using R package ropls (http://www.r-project.org/) [31]. Further, orthogonal projection to latent structures-discriminant analysis (OPLS-DA) [32] is an extension of PLS-DA, which incorporates an Orthogonal Signal Correction (OSC) filter into a PLS model. OPLS-DA was applied in comparison groups using R package models (http://www.r-project.org/). The OPLS-DA model was further validated by cross-validation and permutation test [33]. For cross-validation, the data was partitioned into seven subsets, where each of the subsets was then used as a validation set. R2 indicated the total variation in the data matrix that was explained by the model. Predictive ability (Q2) values represented the most recognized diagnostic statistical parameter to validate the OPLS-DA model in metabolomics. An acceptable predictive model is considered for Q2 value greater than 0.4. and a good predictive model is considered for Q2 value greater than 0.9. Permutation tests randomly permute class labels 200 times and then produce a distribution of R2 values and Q2 values.

### 2.11. Differential Metabolites Analysis

For the targeted metabolomics analysis, we used Student’s *t*-test to screen for significant differences in metabolites between the different comparison groups. When *p* < 0.05, it was determined to be differential metabolites (DMs). To further understand the changes in the abundance of DMs, a volcano plot was constructed for the metabolites screened based on the VIP values and *p*-values, incorporating the FC (fold change) values and *p*-values of the metabolites in the comparison group. The abundance of DMs in the same group was normalized by z-score and then the VIP score of the OPLS-DA was used for visualization. The top 15 metabolites were then drawn and shown in the variable importance in projection (VIP) score plot in descending order [34]. The abundances of DMs were normalized by z-score and hierarchical clustered by R package pheatmap (https://CRAN.R-project.org/package=pheatmap accessed on 15 November 2024) to show the accumulation differences between two groups. To determine the mechanisms of the DMs, Kyoto Encyclopedia of Genes and Genomes [35] was utilized. The DMs were mapped to KEGG metabolic pathways for annotation and enrichment analysis. Pathway enrichment analysis identified significantly enriched metabolic pathways in differential metabolites compared with the whole background. The calculating formula was as follows:

$$P=1-\overset{m-1}{\underset{i=0}{\sum }}\frac{\left(\begin{array}{c} M\\ i \end{array})(\begin{array}{c} N-M\\ n-i \end{array}\right)}{\left(\begin{array}{c} N\\ n \end{array}\right)}$$

Here, *N* is the number of all metabolites that were with KEGG annotation, *n* is the number of DMs in *N*, *M* is the number of all metabolites annotated to specific pathways, and m is number of DMs in *M*. The calculated *p*-value went through FDR correction, taking FDR ≤ 0.05 as a threshold. Pathways meeting this condition were defined as significantly enriched pathways in DMs.

## 3. Results

### 3.1. Mulberry Growth and Yield Parameters Analysis in Response to Differential Stubble Lengths

**Figure 2:**
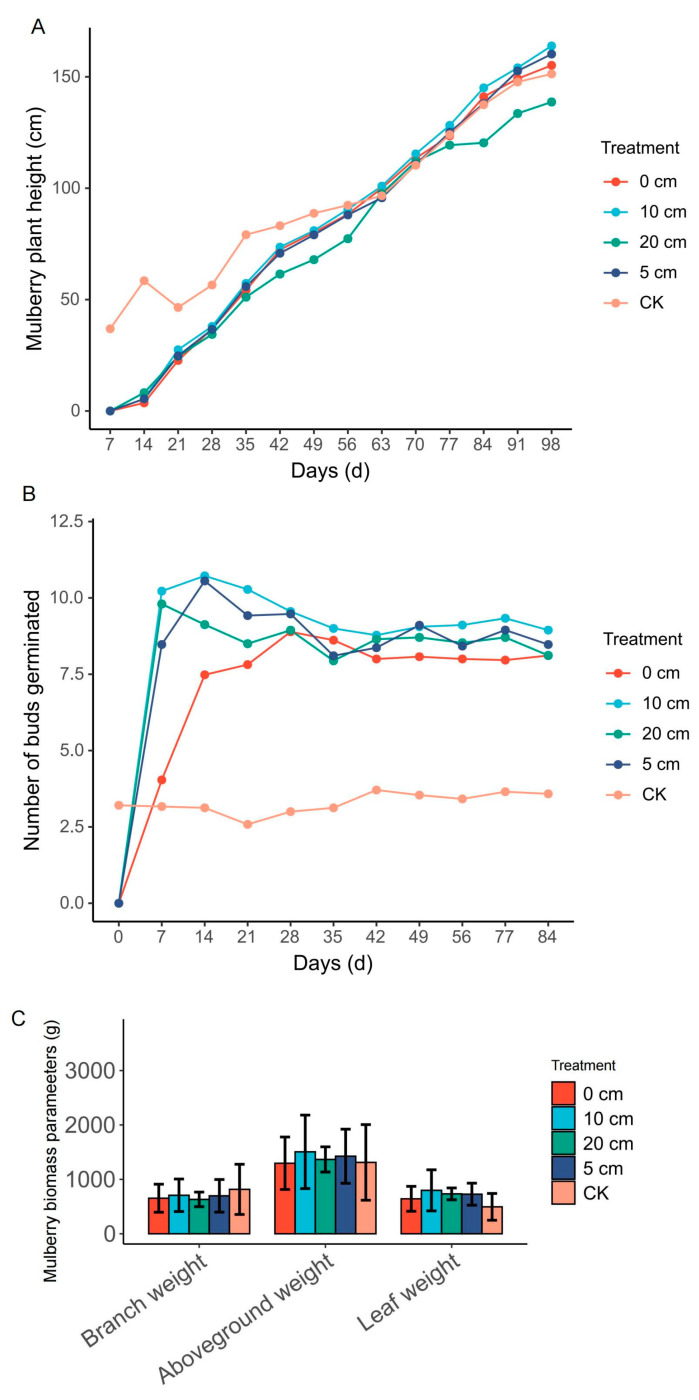
Growth and yield parameters measured after stubble sprout. (**A**) Plant height of regenerated stubble and control. (**B**) Number of buds germinated. (**C**) Biomass. Values are means of ten replicates of plants. CK is unstumped or normal mulberry plants. From 0 cm to 20 cm represents various mulberry stubble lengths.

The growth of *M. alba* after the stubbles were measured in terms of plant height. Seven days after the treatment, the growth in height was significant in the normal plant (CK) compared to the growth of the various stubbles up until the 56th day (Figure 2A). From the 63rd day (9th week), the plants’ height in the stubble was higher, with the highest being recorded in the 0 cm and 10 cm stubble lengths reaching ≈ 95 cm long compared to the CK. After the 9th week, the growth of the 20 cm stubble remained steadily throughout the growing period until the 98th day (14th week). However, the growth of the 0 cm, 5 cm and the 10 cm stubbles outperformed the CK through to the 14th week, with the 10 cm stubble being the best and reaching a height of ≈163 cm (Figure 2A).

Analysis of the germinated buds or yield showed that the stubble plants produced more buds compared to the normal plant (Figure 2B). The number of germinated buds was mostly observed in the first 1 to 2 weeks, with the 10 cm stubble length having the highest number of germinated buds, reaching ≈ 11 in the first week and 9 in the second week. Again, the 5 cm stubble length was second best in terms of the number of buds that germinated. After 3 weeks, the number of buds reduced in all the stubble plants from the 4th week until the growing period, following a down trend in the direction of the normal plant, but the buds’ number remained higher than the normal plant (Figure 2B). Additionally, mulberry yield parameter determination reveal that the aboveground weight and leaf weight were higher in the stubble plants compared to the normal plant, except for the branch weight, which was lower in all the stubble length (Figure 2C). In the stubble groups, the 10 cm stubble produced higher aboveground weight (1500 g), and leaf weight (700 g) compared to the 0 cm, 5cm and the 20 cm (Figure 2C).

### 3.2. Analysis of Photosynthetic Parameters

**Figure 3:**
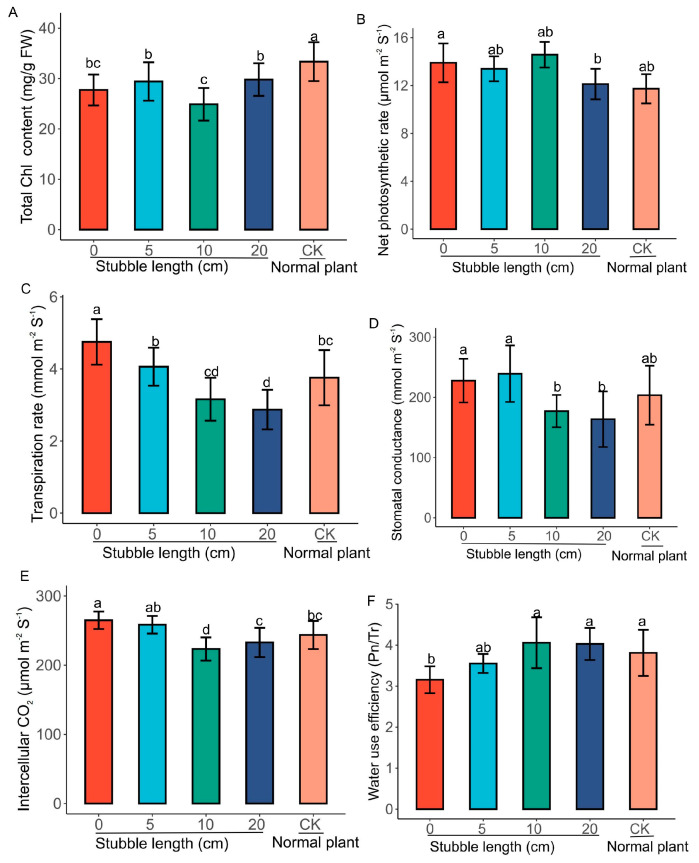
Total chlorophyll and photosynthetic parameters in the differential stubble lengths. (**A**) Total chlorophyl content. (**B**) Net photosynthetic rate (Pn). (**C**) Transpiration rate (Tr). (**D**) Stomatal conductance (Gs). (**E**) Intercellular CO_2_ concentration (Ci). (**F**) Water use efficiency (WUE). Values are means of three replicates of leaf samples. Different letters above the bar represent significant differences (Tukey’s HSD, *p* < 0.05).

Total chlorophyll (Chl) content was significantly lower in the stubble plants compared to the normal plant (Figure 3A). However, the total Chl content decreased in the 10 cm stubble plants compared to the other groups (0 cm, 5 cm and 20 cm), but the total Chl in the 5 cm and 20 cm groups was significantly higher (*p* < 0.05) compared to the 0 cm and the 10 cm. The net photosynthetic rate (Pn) levels were increased in the stubble plants compared to the normal plant (Figure 3B). The Pn levels in the 10 cm and 0 cm stubbles were slightly higher than the 5 cm and the 20 cm groups. The transpiration rate (Tr) increased in the 0 cm stubble plants and significantly differed from the other stubbles and the normal plants (Figure 3C). The Tr in the 10 cm and 20 cm stubbles were significantly lower (*p* < 0.05) compared to the 0 cm and the 5 cm stubbles. A similar trend was found in the stomatal conductance (Gs) **(**Figure 3D), and the intercellular CO_2_ concentration (Ci) (Figure 3E), where the Gs and Ci were significantly reduced (*p* < 0.05) in the 10 cm and 20 cm groups. Further, the water use efficiency (WUE) level was higher in the 5 cm, 10 cm and 20 cm stubbles compared to the 0 cm stubbles (Figure 3F). However, no significant difference (*p* < 0.05) was found among the 10 cm, 20 cm stubbles and the normal plants.

### 3.3. Analysis of the Elemental and Crude Protein Contents in Stubble Plants

**Figure 4:**
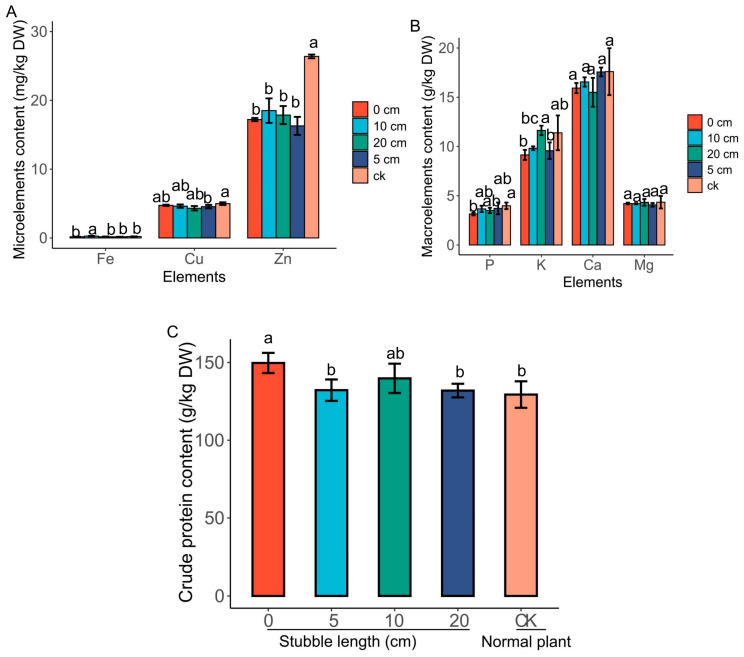
Elemental and crude protein contents in stubble plant leaves. (**A**) Microelement content. (**B**) Macroelement content. (**C**) Crude protein content. Values are means of three replicates of leaf samples. Different letters above the bar represent significant differences (Tukey’s HSD, *p* < 0.05).

The quality of the mulberry leaves was determined by analyzing the nutritional contents of the leaves from the stubble plants in comparison to the CK. Among the microelements content, zinc (Zn) content was significantly higher (*p* < 0.05) in the normal plant (Figure 4A). Meanwhile Zn content was slightly higher in the 10 cm stubbles compared to the 0 cm, 5 cm and the 20 cm stubbles, but they were statistically not different. Copper content in the stubble plants was almost the same in the normal plant. However, iron (Fe) content was very low in all the stubbles, including those of the normal plant. As shown in Figure 4B, among the macronutrients, calcium (Ca) content was higher in all stubbles, including those of the normal plant, with the 5 cm stubbles and the CK reaching 17.7 g/kg. However, the Ca content in the stubble plants was significantly different from the normal plant. Again, potassium (K) content was increased in the stubble plants as well as in the normal plant; however, the K content in the normal plant was significantly different from the stubble plants (Figure 4B). Meanwhile, magnesium (Mg) and phosphorus (P) contents were very low compared to Ca and K in the stubble and normal plants. Analysis of the crude protein content showed that the crude protein was higher in the stubble plants compared to the normal plant, especially in the 0 cm group, reaching about 149 g/kg, followed by the 10 cm group, reaching 147 g/kg. However, the 5 cm and 20 cm stubbles were not significantly different from the normal plant (CK).

### 3.4. Biochemical Analysis of Some Major Endogenous Plant Hormones Contents and Correlation Analysis

**Figure 5:**
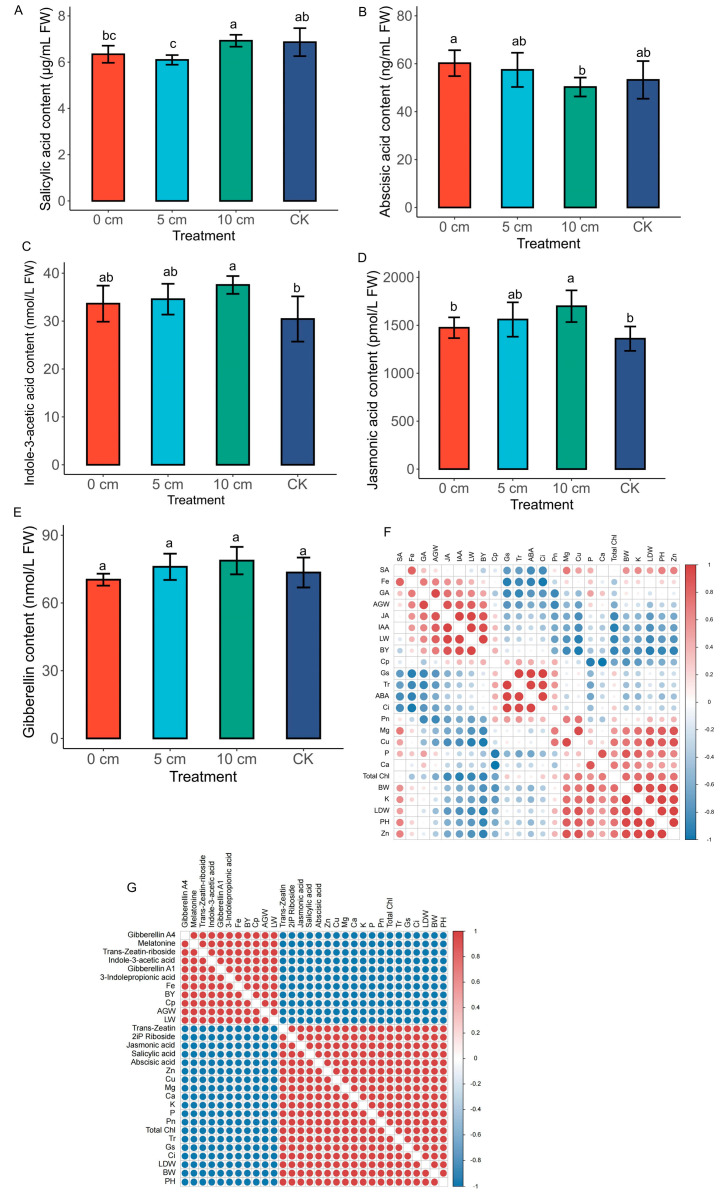
Biochemical analysis of some major plant hormone contents and correlation analysis of elements, metabolites, growth and photosynthetic parameters. (**A**) Salicylic acid (SA) contents. (**B**) Abscisic acid (ABA) contents. (**C**) Indole-3-acetic acid (IAA). (**D**) Jasmonic acid (JA). (**E**) Gibberellin (GA). (**F**) Pearson correlation coefficient involving hormones measured by biochemical means, elements, growth and photosynthetic parameters. (**G**) Pearson correlation coefficient involving differential metabolites (DMs), elements, growth and photosynthetic parameters. Red and blue colors in the correlation plots indicate positive and negative correlation, respectively. BW (branch weight), AGW (aboveground weight), LDW (leaf dry weight), PH (plant height) Pn (net photosynthetic rate), LW (leaf weight), BY (bud yield), Cp (crude protein), Ci (intercellular CO_2_ concentration), Tr (transpiration rate), Gs (stomatal conductance), Zn (zinc), Fe (iron), Cu (copper), Ca (calcium), K (potassium), Mg (magnesium), P (phosphorus). Values are means of three replicates of leaf samples. Different letters above the bar represent significant differences (Tukey’s HSD, *p* < 0.05).

Plant hormones, including salicylic acid (SA), abscisic acid (ABA), indole-3-acetic acid (IAA), jasmonic acid (JA), and gibberellin (GA) were evaluated in stubble (0 cm, 5 cm, 10 cm) plants relative to the normal plant (CK). As shown in Figure 5A, SA content was higher (7 µg/mL) in the 10 cm stubble length and the CK compared to the 0 cm and the 5 cm stubble lengths. Also, ABA content was higher in the 0 cm (61 ng/mL) and 5 cm (58 ng/mL) stubble lengths compared to the CK, with the 10 cm stubble length recording (50 ng/mL) the lowest (Figure 5B). However, the IAA content in the stubble plants was higher than the CK plant (Figure 5C). The content of IAA was significantly higher (*p* < 0.05) in the 10 cm stubble (38 nmol/L) compared to the 0 cm and the 5 cm stubbles. A similar pattern was observed in the JA content, where JA content in the 10 cm stubble was significantly higher than in the 0 cm and 5 cm stubbles and the CK (Figure 5D). Again, GA content was higher in the 10 cm and 5 cm stubbles, with the 10 cm stubble being the best, but lower in the 0 cm stubble and the CK. Interestingly, there was no significant difference (*p* < 0.05) in the stubbles (Figure 5E). The ratios of the phytohormones were analyzed (Table S1). From the results, the ratio of the ABA and GA (ABA:GA) reveals the highest proportion including 0.73, 0.86, 0.76 and 0.64 in CK, 0 cm, 5 cm and 10 cm stubbles, respectively. Again, GA and JA (GA:JA) showed below average ratios of 0.05, 0.048, 0.049 and 0.046 in CK, 0 cm, 5 cm and 10 cm stubbles, respectively. A similar trend was observed in IAA:GA ratio of 0.414 in the CK, 0.478 in 0 cm, 0.455 in the 5 cm and 0.478 in the 10 cm were recorded (Table S1). The ratios of IAA and ABA (IAA:ABA) recorded above average ratios of 0.572, 0.558, 0.602, and 0.748 in CK, 0 cm, 5 cm and 10 cm stubbles, respectively.

An intriguing correlation analysis was performed using the hormones identified biochemically and the differentially expressed hormones identified by the metabolomics analysis as well as the growth, nutritional contents and photosynthetic parameters. As illustrated in Figure 5F, SA correlated positively with elements such as Fe, K, Mg, Zn (r = 1), and P, Cu (r = 0.8). Again, SA significantly and positively correlated with BW (branch weight, r = 0.89) and AGW (aboveground weight, r = 0.5), LDW (leaf dry weight, r = 0.87), PH (plant height, r = 1) and Pn (net photosynthetic rate, r = 0.34). Furthermore, GA positively correlated significantly with Fe (r = 1), Ca (r = 0.35), P (r = 0.5), LW (leaf weight, r = 0.85), BY (bud yield, r = 0.62), and AGW (r = 1). Several parameters, including Cp (crude protein), BY, LW, AGW and Fe positively correlated with JA and IAA (Figure 5F). Also, ABA correlated positively with Cp (r = 0.45), Gs (stomatal conductance, r = 1), Tr (transpiration rate, r = 1), Pn (net photosynthetic rate, r = 0,45) and Ci (intercellular CO_2_ concentration, r = 1). As depicted in Figure 5G, metabolites such as 3-indolepropionic acid, gibberellin A1, gibberellin A1, Indole-3-acetic acid, trans-zeatin-riboside, and melatonin identified from the metabolomics analysis correlated strongly and positively (r = 1) with Fe, BY, Cp, AGW and LW. On the other hand, Trans-zeatin, 2iP riboside, jasmonic acid, salicylic acid and abscisic acid correlated positively and strongly with Zn, Cu, Mg, Ca, K, P, Pn, total Chl, Tr, Gs, Ci, LDW, BW and BH (r = 1) (Figure 5G).

### 3.5. Phytohormones Profile of Leaves of M. alba Differential Stubble Lengths

**Figure 6:**
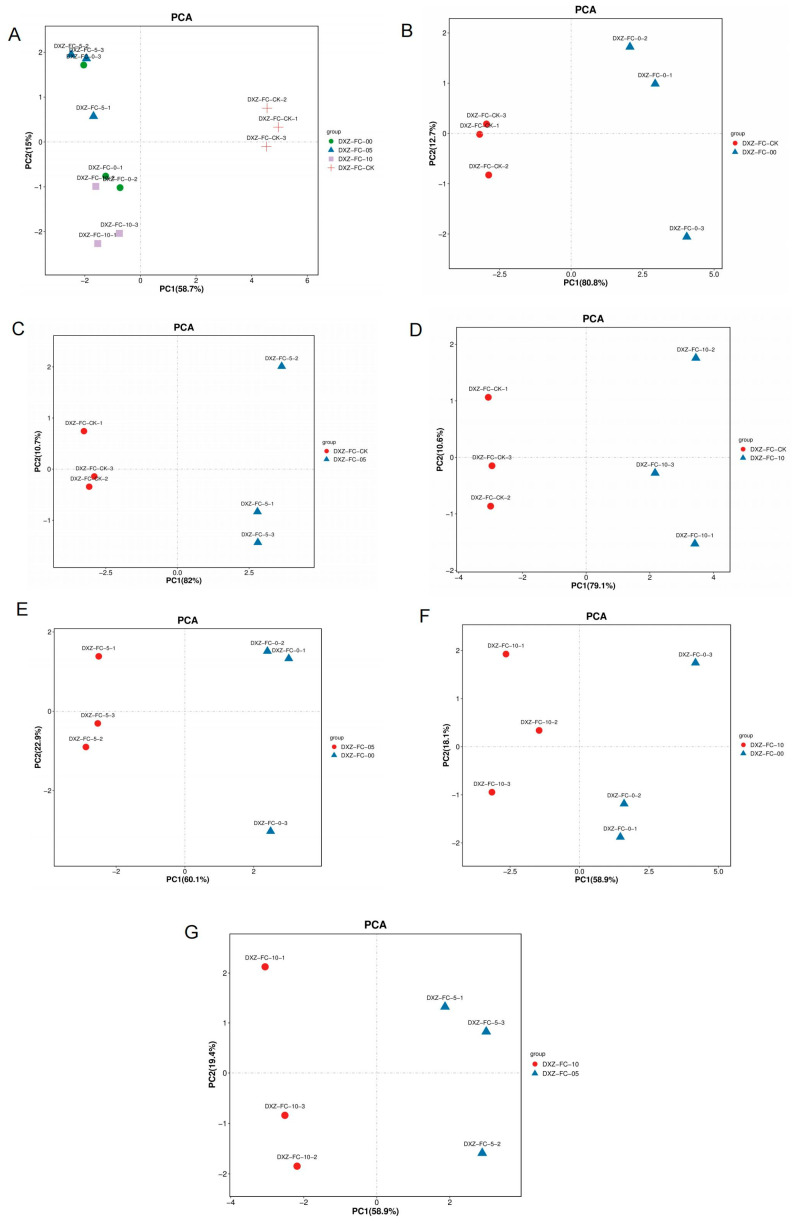
Principal component analysis (PCA) of the metabolites in samples. (**A**) PCA of metabolites in all sample groups. (**B**–**D**) PCA analysis comparing the stubbles to only the control plants as follows: DXZ-FC-CK-vs-DXZ-FC-0; DXZ-FC-CK-vs-DXZ-FC-5; DXZ-FC-CK-vs-DXZ-FC-10. (**E**–**G**) PCA analysis comparisons within and among the stubbles as follows: DXZ-FC-5-vs-DXZ-FC-0, DXZ-FC-10-vs-DXZ-FC-0, and DXZ-FC-10-vs-DXZ-FC-5, respectively. DXZ-FC-CK; unstumped or normal mulberry plant. DXZ-FC-0; 0 cm stubble. DXZ-FC-5; 5 cm stubble. DXZ-FC-10; 10 cm stubble.

**Figure 7:**
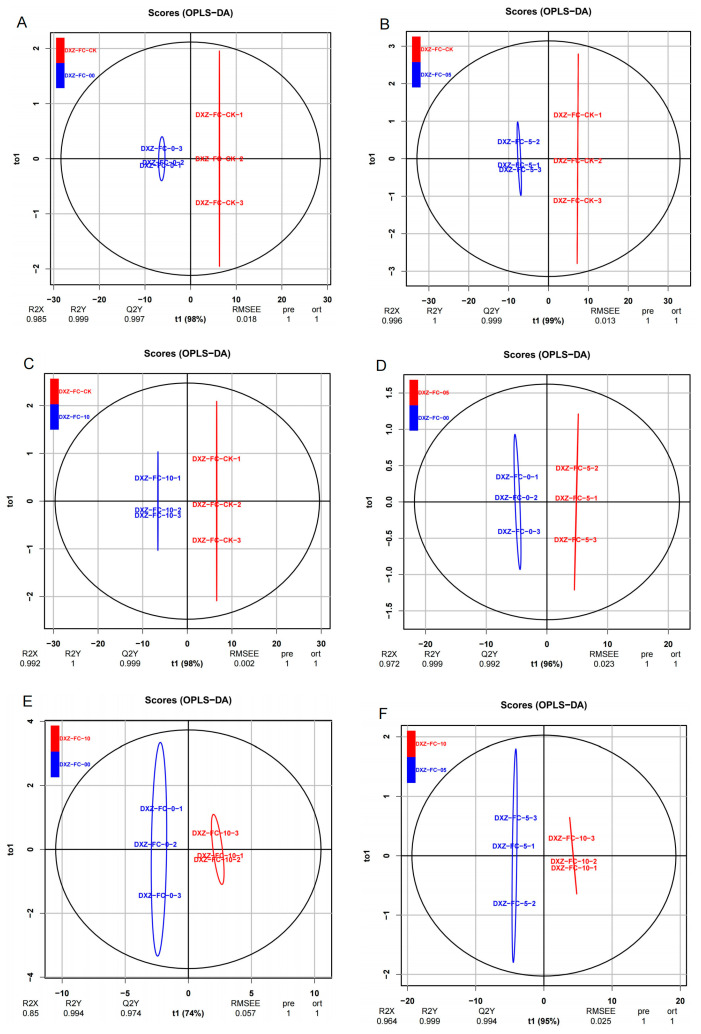
Orthogonal partial least-squares-discriminant analysis (OPLS-DA) of metabolites in samples. (**A**–**C**) OPLS-DA analysis comparing the stubbles to only the control plants as follows: DXZ-FC-CK-vs-DXZ-FC-0; DXZ-FC-CK-vs-DXZ-FC-5; DXZ-FC-CK-vs-DXZ-FC-10. (**D**–**F**) OPLS-DA analysis comparing the stubbles to only the control plants as follows: DXZ-FC-5-vs-DXZ-FC-0, DXZ-FC-10-vs-DXZ-FC-0, and DXZ-FC-10-vs-DXZ-FC-5.

To analyze the phytohormones profile of *M. alba* at the differential stubble lengths, we performed a targeted metabolomics analysis using leaves from 0 cm (DXZ-FC-0), 5 cm (DXZ-FC-5), and 10 cm (DXZ-FC-10) mulberry stubbles, as well as normal mulberry plants, designated as CK (DXZ-FC-CK). From the targeted metabolomics analysis results, a total of 19 phytohormones were expressed from leaves sampled from the differential stubble length (Table S2). The obtained metabolites (hormones) were subjected to multivariate analysis such as principal component analysis (PCA), partial least squares discriminant analysis (PLS-DA), and orthogonal partial least squares discriminant analysis (OPLS-DA). As indicated in Figure 6, the PCA plot showed a total variation of 73.7% in the two PCAs, with PC1 explaining 58.7% in all samples (Figure 6A). Again, PCAs regarding different group comparisons reveal variation including 93.5% in DXZ-FC-CK-vs-DXZ-FC-0, 92.7% in DXZ-FC-CK-vs-DXZ-FC-5, 89.7% in DXZ-FC-CK-vs-DXZ-FC-10, 83% in DXZ-FC-5-vs-DXZ-FC-0, 77% in DXZ-FC-10-vs-DXZ-FC-0, and 78.3% in DXZ-FC-10-vs-DXZ-FC-5 (Figure 6B–G). Further, the OPLS-DA score plot showed that 98%, 99%, 98%, 96%, 74% and 95% variations occurred between DXZ-FC-CK-vs-DXZ-FC-0, DXZ-FC-CK-vs-DXZ-FC-5, DXZ-FC-CK-vs-DXZ-FC-10, DXZ-FC-5-vs-DXZ-FC-0, DXZ-FC-10-vs-DXZ-FC-0 and DXZ-FC-10-vs-DXZ-FC-5, respectively (Figure 6A–F). The following Q2 values of the predictive model were observed: 0.997 (p-value; 0.018) in DXZ-FC-CK-vs-DXZ-FC-0, 0.999 (*p*-value; 0.013) in DXZ-FC-CK-vs-DXZ-FC-5, and 0.999 (*p*-value; 0.002) in DXZ-FC-CK-vs-DXZ-FC-10 (Figure 7A–C). In the inter-group comparison, the following values were observed: 0.992 (*p*-value; 0.023) in DXZ-FC-5-vs-DXZ-FC-0, 0.974 (*p*-value; 0.057) in DXZ-FC-10-vs-DXZ-FC-0 and 0.994 (*p*-value; 0.025) in DXZ-FC-10-vs-DXZ-FC-5 (Figure 7D–F). As compared to the PCA, the OPLS-DA score plot displayed a more distinct clustering pattern, indicating metabolite differences between the normal plants and the stubble lengths. This signifies the reliability of the discrimination model. Figure S1 reveals the permutation testing of the OPLS-DA score distributions.

### 3.6. Differential Metabolites Analysis (DMs) of Leaves of M. alba Differential Stubble Lengths

**Figure 8:**
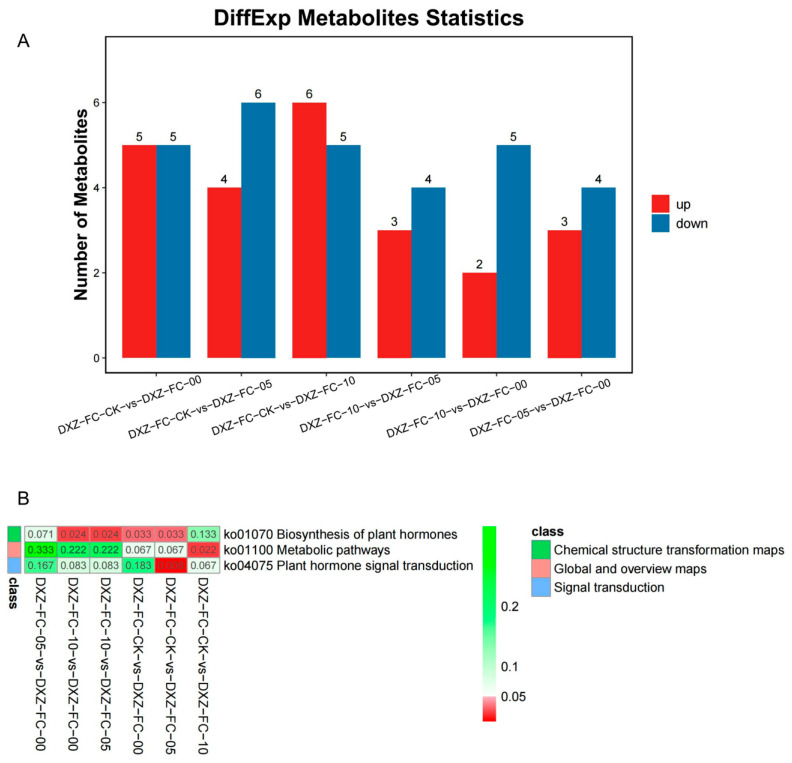
Statistics of the differential metabolites (DMs) from group comparison. (**A**) Bar plot indicating the up- and down-regulation of the DMs. The red and blue colors indicate upregulated and downregulated DMs from group comparison. (**B**) The DMs involved in the significant KEGG pathways. The deep red to deep green color denotes the level of significant (*p* < 0.05). Class represents the classification of the pathways.

**Figure 9:**
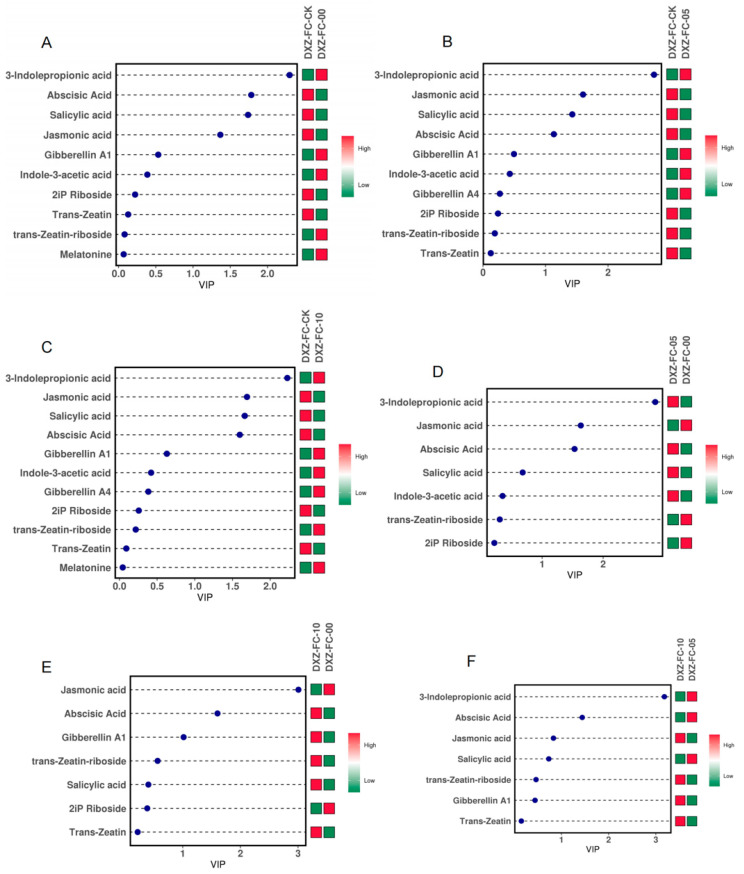
The DMs and the variable importance in projection (VIP) values. (**A**–**F**) VIP values of differential metabolites in DXZ-FC-CK-vs-DXZ-FC-0, DXZ-FC-CK-vs-DXZ-FC-5, DXZ-FC-CK-vs-DXZ-FC-10, DXZ-FC-5-vs-DXZ-FC-0, DXZ-FC-10-vs-DXZ-FC-0, and DXZ-FC-10-vs-DXZ-FC-5, respectively. The x-axis represents the VIP value, the left side of the y-axis represents the DMs name, and the right side represents the DMs concentrations (the red color, high concentration; green color, low concentration).

The analysis of the differential metabolites (DMs) showed that 10 DMs comprising 5 up- and 5 downregulation were observed in the DXZ-FC-CK-vs-DXZ-FC-0, 10 DMs (4 up and 6 downregulated) in the DXZ-FC-CK-vs-DXZ-FC-5, and 11 DMs (6 up- and 5 downregulated) in the DXZ-FC-CK-vs-DXZ-FC-10 (Figure 8A). Further comparative analysis of the DMs was carried out among the stubble groups. From the results, 7 DMs (3 up- and 4 downregulated) were in the DXZ-FC-10-vs-DXZ-FC-5 comparison, 7 DMs comprising 2 up- and 5 downregulated in the DXZ-FC-10-vs-DXZ-FC-0 comparison, and finally 7 DMs made up of 3 up- and 4 downregulated in the DXZ-FC-5-vs-DXZ-FC-0 comparison (Figure 8A). Metabolites, including 3-indolepropionic acid, gibberellin A1, indole-3-acetic acid, gibberellin A4, melatonin, and trans-zeatin-riboside were upregulated in either DXZ-FC-CK-vs-DXZ-FC-0, DXZ-FC-CK-vs-DXZ-FC-5 or DXZ-FC-CK-vs-DXZ-FC-10 (Table 1). Moreover, downregulated DMs such as trans-zeatin, 2iP riboside, abscisic acid, salicylic acid, and jasmonic acid were downregulated in these stubble plants (Table 1). Volcano plots depicting the distribution of DMs were analyzed and the results reveal that most of the DMs’ distribution was above the threshold (Figure S2). The influence of the DMs in the stubble sample differentiation using the VIP values was noted. From the results, metabolites, such as abscisic acid, salicylic acid, and jasmonic acid, 3-indolepropionic acid, and gibberellin A1 were influential (Figure 9).

### 3.7. Hierarchical Cluster Analysis of Differential Metabolites (DMs)

**Figure 10:**
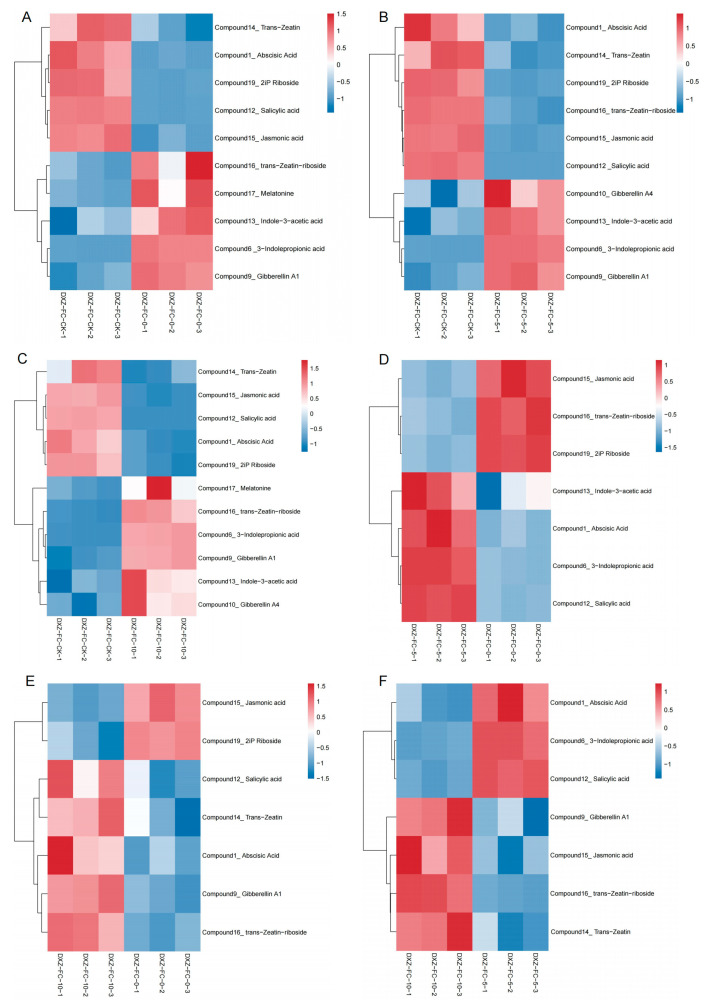
Hierarchical clustering analysis of the DMs. (**A**–**F**) Pattern heat map of DMs in DXZ-FC-CK-vs-DXZ-FC-0, DXZ-FC-CK-vs-DXZ-FC-5, DXZ-FC-CK-vs-DXZ-FC-10, DXZ-FC-5-vs-DXZ-FC-0, DXZ-FC-10-vs-DXZ-FC-0, and DXZ-FC-10-vs-DXZ-FC-5, respectively. The red color indicates high concentration, and the blue color indicates low concentration.

Hierarchical cluster analysis (HCA) highlighted similar expression patterns between the same stubble length groups and variations between different stubble groups (Figure 10). Metabolites, including jasmonic acid, salicylic acid, 2iP riboside, and abscisic acid were lower in concentration in the 0 cm, 5 cm, and 10 cm stubble lengths, but these compounds with higher concentration in the normal mulberry plant (Figure 10A–C). However, metabolites such as gibberellin A1, melatonin, indole-3-acetic acid, 3-indolepropionic acid, gibberellin A4 and trans-zeatin-riboside contents were high in the 0 cm, 5 cm, or 10 cm stubble length but less in the normal plant. Trans-zeatin-riboside content was lower in the 5 cm stubble length compared to the 0 cm and 10 cm stubble lengths.

### 3.8. KEGG Classification and Enrichment Analysis of Differential Metabolites

**Figure 11:**
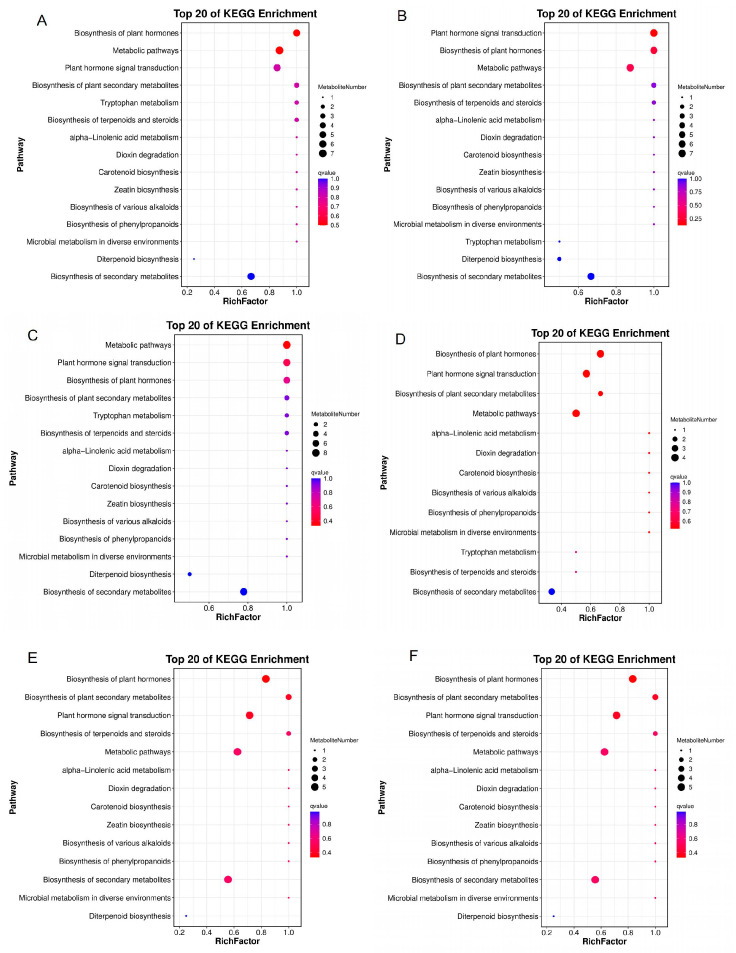
KEGG pathway enrichment analysis of the DMs. (**A**–**F**) Bubble plot showing the top 20 KEGG pathways in DXZ-FC-CK-vs-DXZ-FC-0, DXZ-FC-CK-vs-DXZ-FC-5, DXZ-FC-CK-vs-DXZ-FC-10, DXZ-FC-5-vs-DXZ-FC-0, DXZ-FC-10-vs-DXZ-FC-0, and DXZ-FC-10-vs-DXZ-FC-5, respectively. Bubble size indicates the number of DMs. The bubble color from blue to red indicates an increase in significance (q-value).

Analysis of the pathway classification and enrichment involving the DMs were performed using the Kyoto Encyclopedia of Genes and Genomes (KEGG) database. From the results, the DMs were annotated to three main KEGG pathways, including metabolism, environmental information processing and signal transduction (Figure S3). Further, the DMs were significantly (*p* ≤ 0.05) classified into three pathways, including chemical structure transformation maps (biosynthesis of plant hormones), global and overview maps (metabolic pathways), and signal transduction (plant hormone signal transduction) (Figure 8B). The top 20 KEGG enrichment analyses reveal that the DMs were enriched in several pathways, including zeatin biosynthesis, plant hormone signal transduction, metabolic pathways, biosynthesis of plant hormones, dioxin degradation, biosynthesis of secondary metabolites, and many other (Figure 11A–F).

**Figure 12:**
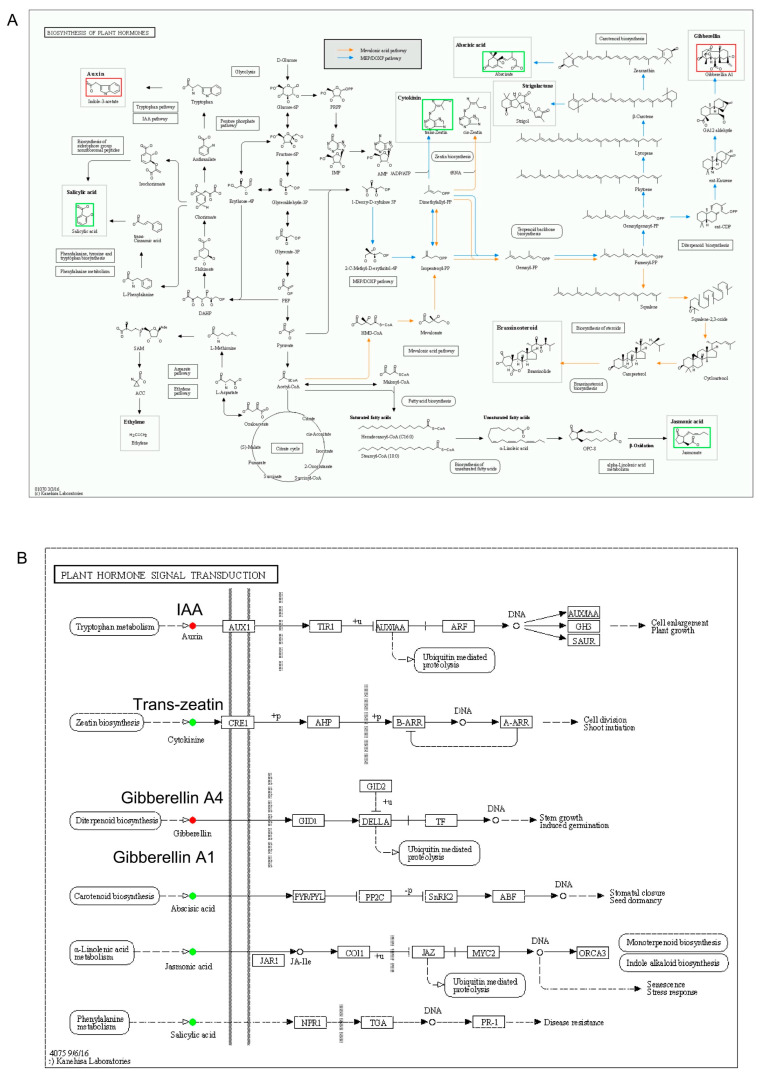
KEGG mechanisms pathways based on significant pathways (*p* < 0.05) with the DMs. (**A**) Biosynthesis of plant hormones pathway. (**B**) Plant hormone signal transduction pathway. Red denotes upregulated DMs, and green denotes downregulated DMs in the pathways.

In the key significant pathways, biosynthesis of plant hormones (ko01070) was significant (*p* ≤ 0.05) in all the stubbles comparisons except in the 10 cm stubble length. In this pathway, metabolites, including IAA and gibberellin A1 (all upregulated), as well as SA, JA, ABA and trans-zeatin (all downregulated), were implicated in this pathway (Figure 12A). Also, the metabolic pathways (ko01100) were significant only in the 10 cm stubble treatment, and this pathway was enriched with several metabolites, such as ABA, SA, JA, and trans-zeatin (all downregulated), as well as gibberellin A1, gibberellin A4, IAA and melatonin (all upregulated). The plant hormone signal transduction pathway (ko04575) was only significant in the 5 cm stubble treatment and enriched with metabolites such as IAA, gibberellin A1, and gibberellin A4 (all upregulated), as well as ABA, SA, JA, and trans-zeatin (all downregulated) (Figure 12B).

## 4. Discussion

The sericulture industries around the globe rely exclusively on *Morus* sp to primarily feed the domesticated mulberry silkworm (*Bombyx mori* L.). This makes the leaves of mulberry the single most important part of the mulberry plants. Multiple efforts towards the intensification and mass production of mulberry leaves are increased exponentially to bridge the production gaps industries encounter in the feeding of the silkworm during the larvae stage. These intensification and mass production strategies depend largely on the propagation or cultivation method, or the system employed. Mulberry is reproduced via both sexual and vegetative means. However, the vegetative or asexual means of propagation are preferably the most adopted due to multiple complications, including availability of fruit, drudgery in fruit collection, poor germination percentage, less storage period, time-consuming mulberry sapling production, late maturity (takes about a decade to bear fruits from seeds), high heterozygosity, and poor viability associated with the sexual propagation method [36,37]. This has made the vegetative propagation method the most pivotal and commonly used means by the commercial producers of cocoons from silkworms. The planting of mulberry via sapling methods such as grafting, budding, and layering due to their ability to exponentially multiply lateral buds on stem cuttings that enhance rapid shoot formation by increasing the rate of cell division and promoting the vigor of the sprouted cuttings is crucial [36].

Now, the sprouting of mulberry using stubbles is a hotspot method in the rapid and mass production of mulberry leaves. We speculated that differential stubble lengths not only promote rapid growth and improve physiological traits but also alter the synthesis and accumulation of endogenous plant hormones, which have been reported to modulate the formation of adventitious roots and growth of mulberry [16,38].

We carried out a series of analyses to check whether the differential stubble lengths from mulberry plants promote early growth in comparison to fully established mulberry plants (control). Our hypothesis and curiosity resulted in revealing some striking results heightening the premise that the differential stubble lengths enhance rapid growth of mulberry plants compared to the control. The use of differential stubble lengths of mulberry promoted growth, bud formation, and biomass (Figure 2A–C), highlighting the rapid shoot formation by increasing the rate of cell division and promoting the vigor of the sprouted cuttings obtained from stubbles. The highest growth and the associated parameters were recorded in 10 cm stubble length (Figure 2A–C), indicating the influence of differential stubble lengths on the growth of mulberry. This result was supported by the physiological and photosynthetic-related traits, where the results consistently heightened the fact that the differential stubble lengths promoted higher growth and production in mulberry relative to the control. For instance, net photosynthesis, transpiration rate, stomatal conductance, and intercellular CO_2_ levels were generally observed to be relatively higher in 0 cm and 5 cm stubble lengths in comparison to the control (Figure 3B–D), suggesting that the increased growth recorded in the stubble is in accordance with the other growth determinants. It is quite plausible to conclude that the growth, developments, and rapid production of leaves depend not only on the type of cuttings, environmental condition, age, genotype, etc., but also rely on the differential stubble lengths, as highlighted and established in this study.

Referring to our current results and other prior studies, employing stubbles as cutting techniques has manifested some promising signatures in ameliorating mulberry survival rates by improving the sprouting of new shoots from buds. However, the exogenous application of growth regulators has auspicious signs of expediting and improving critical processes such as cell division, rhizome elongation, and shoot sprouting from buds [39,40]. For example, a study conducted by Zhang et al. (2017) [38] revealed that the exogenous application of IAA, naphthalene acetic acid (NAA), or green growth regulator (GGR) to *M*. *hupehensis* improved adventitious root at different stages, concomitantly shortened rooting time by 25–47.4%, and promoted the rooting efficiencies of cuttings by 0.9–1.3 times, as compared to the control, indicating that fortification of soil with growth hormones expedites growth and rapid production of shoots. Even though we supplied no exogenous plant growth-enhancing hormones to the stubbles after cutting, we speculated that the termination of plant growth by cutting the shoots to obtain stubbles would cause external shocks that could trigger the de novo synthesis of plant-growth-enhancing hormones or instantaneous synthesis and modulation of plant growth regulators that ensure early sprouting of new juvenile and succulent shoots in mulberry. This hypothesis was narrowed down to focus on a more specific question: at what stubble length does sprouting of new shoots from buds and growth concomitantly elevate the induction and accumulation of plant-growth-enhancing hormones in mulberry? Intriguingly, our findings revealed striking observations that the morphological, physiological, biochemical, and targeted metabolomics signature metamorphosis associated with early sprouting and growth complemented the modulation of endogenous plant growth hormones and regulators. These attestations are manifested in the numerous significant and positive correlations exhibited among the morphological (plant height, aboveground weights, number of buds, branch and leaf weights), physiological (photosynthetic parameters, elemental analysis), biochemical (chlorophyll content, crude protein, phytohormones), and targeted metabolomics (differentially accumulated phytohormones) determinants in our study. For example, striking evidence from our study revealed that endogenous SA determined via biochemical analysis exhibited highly significant and positive associations with branch weight (r = 0.89), aboveground weight (r = 0.5), leaf weight (r = 0.87), plant height (r = 1) and net photosynthetic rate (r = 0.34) (Figure 5F), highlighting the role of SA in the sprouting and rejuvenation of stubbles used as a vegetative propagation technique for the rapid production of mulberry leaves for sericulture industries. In other developments, the biochemical analysis of endogenous GA content in leaves of mulberry presented yet another highly significant and positive correlation with determinants, including Fe, Ca (r = 0.35), P (r = 0.5), leaf weight (r = 0.85), bud yield (r = 0.62) and aboveground weight (r = 1) (Figure 5F), confirming that GA was crucially involved in the sprouting and early growth of mulberry stubbles by enhancing cell division and elongation [41]. Further multiple findings from our results saw crude protein, bud yield, leaf weight, aboveground weight, and Fe significantly and positively correlating with biochemically determined JA and IAA (Figure 5F), highlighting that endogenous JA and IAA play a pivotal role by improving the cell regeneration abilities of mulberry stubbles. Meanwhile, ABA, tagged as an inhibitory plant hormone, observed interesting synergistic interactions by correlating positively with crude protein (r = 0.45), stomatal conductance (r = 1), transpiration rate (r = 1), net photosynthetic rate (r = 0.45) and intercellular CO_2_ concentration (r = 1) (Figure 5F). This phenomenon could suggest that endogenous ABA plays a crucial role in stubble regeneration and sprouting. Similarly, it has previously been reported that the ABA content of vegetable and mulberry cuttings was found to increase assistance in regulating the adaptability of cuttings to stress by hardening them for the formation of root primordia [42]. To further confirm our findings from the biochemical analysis ascertaining that phytohormones play a relevant role in stubble rejuvenation in mulberry plants, we correlated the differentially accumulated metabolites identified from our targeted metabolomics analysis of phytohormones with growth-related and other physiological parameters. Remarkable correlations, including 3-indolepropionic acid, gibberellin A1, gibberellin A1, Indole-3-acetic acid, trans-zeatin-riboside, and melatonin identified from the metabolomics analysis, strongly correlated positively with Fe content, bud yield, crude protein, leaf weight, and aboveground weight (Figure 5G). The above multiple findings and pieces of evidence suggest that the stimulation and modulation of endogenous hormones in mulberry improved the regeneration and growth of stubbles, supporting the hypothesis that stubble growth is greatly enhanced by endogenous hormones.

The differential stubble lengths not only promoted growth and regeneration of mulberry cuttings, but also regulated the various hormones implicated in the biosynthesis of the plant hormones (ko01070) pathway. In this study, the remarkable enrichment and upregulation of the biosynthesis of the plant hormones pathway, generally in all stubbles, except the 10 cm stubble, indicate an alteration to the pathway triggered by the differential stubble lengths in mulberry plants (Figure 12A; Table 1). The stubble rhizogenesis modulated endogenous phytohormones and significantly altered the biosynthesis of plant hormones by modulating the upregulation of IAA and gibberellin A1 in mulberry leaves (Figure 11A). The upregulation of IAA was further supported by the increased IAA content recorded in the 10 cm stubble (Figure 5C), which tandemly correlated positively with crude protein, bud yield, leaf weight, aboveground weight, and Fe (Figure 5F), signaling that IAA was directly involved in the sprouting of stubbles. IAA is associated with adventitious root induction, tuber expansion by triggering the formation of a tuber layer and doubling xylem cell proliferation [26]. IAA biosynthesis commences with its precursor, tryptophan, that undergoes a multiple chemical metamorphosis via decarboxylation to form tryptamine, which is subsequently changed through direct oxidative deamination to IAA. Interestingly, tryptophan metabolism was found to be significantly enriched in our study (Figure 11), further augmenting the upregulation of IAA and confirming the influence of differential stubble length on mulberry growth and induction of endogenous hormones.

GA biosynthesis is crucial in the hormone biosynthesis pathway. This process occurs through three catalytic stages, where geranylgeranyl diphosphate is catalyzed by ent-copalyl diphosphate synthase to form ent-kaurene. The ent-kaurene is oxidized to GA12-aldehyde by ent-kaurene oxidase, which is composed of GA precursor. The GA12-aldehyde is further activated by cytochrome P-450 mono-oxygenases to enter the cytosol of the cell, where it is catalyzed by 2-oxoglutarate-dependent dioxygenases [43]. The biosynthesis of GA propels plants to stimulate shoot growth, the extension of the internode, flowering, stem growth, seed germination, fruit setting, and the inhibition of the formation of free radicals, which induce lipid peroxidation [44]. Our study identified significant upregulation of two GA isoforms, including Gibberellin A1 and Gibberellin A4 in DXZ-FC-CK-vs-DXZ-FC-0, DXZ-FC-CK-vs-DXZ-FC-5 and DXZ-FC-CK-vs-DXZ-FC-10 (Table 1), and significantly altered the hormone biosynthesis pathway in mulberry leaves (Figure 12A). This is a strong testimony to its crucial role in the sprouting of stubble by stimulating higher shoot growth and stem growth [45] in this study. To further buttress our findings, the high accumulation of GA not only had a direct effect on mulberry growth by increasing GA content in, for example, 10 cm and 5 cm stubbles relative to the control (Figure 5E), but also had a direct and positive correlation with all growth and photosynthetic parameters (Figure 5F). This indicates that the higher GA biosynthesis promoted shoot growth by enhancing rapid sprouting and early growth of mulberry stubbles. Although we supplied no exogenous GA, exogenous GA_3_ was found to have regulated the biosynthesis of phytohormones and plant hormone signal transduction pathways of endogenous phytohormones, which modulates the plant growth in sweet cherry and *Pseudostellaria heterophylla* [46,47]. Of course, contradictory responses and antagonistic interactions among phytohormones were highly expected and could not be ruled out entirely. We found that the regulation of the plant hormone biosynthesis pathway by the different stubble lengths concurrently triggered downregulation of phytohormones such as SA, JA, ABA, and trans-zeatin (Figure 12A), which are known to be involved in signaling. The up- and down-regulations observed, respectively, in gibberellin A1, gibberellin A4, and ABA in this pathway show an antagonistic relationship existing between gibberellin A1, and ABA.

Although both JA and SA play pivotal roles in many physiological processes in plant growth and development, they are known to be keenly involved in signaling through the mediation of plant responses to biotic and abiotic stresses [48,49], making them indispensable constituents in the plant hormone signal transduction pathways. In our study, a significantly altered and modulated expression occurred in the plant hormonal signal transduction pathway, and this was complemented by the downregulation of phytohormones, including ABA, SA, JA, and trans-zeatin (Figure 12B). However, the same pathway saw the upregulation of phytohormones such as IAA, gibberellin A1, and gibberellin A4 in the 5 cm stubble, which we hypothesized to be associated with unique regulation of the biosynthesis of both the plant hormonal biosynthesis and signal transduction pathways in mulberry plants. Meanwhile, the absolute downregulation of ABA, SA, and JA in stubble comparisons such as DXZ-FC-CK-vs-DXZ-FC-0, DXZ-FC-CK-vs-DXZ-FC-5, and DXZ-FC-CK-vs-DXZ-FC-10 (Table 1) further supports our speculation and could suggest that mulberry plants repress expression and accumulation of ABA, SA, and JA and instantaneously increase the contents of GA (gibberellin A1, gibberellin A4), IAA and melatonin to balance its metabolic adjustment; it could also be a sign that an alternative signal transduction route has been employed by the mulberry. A similar finding was reported in *Camellia sinensis* exposed to MT and GA [50]. It is quite plausible to conclude that the use of different stubble lengths promotes growth and regulates plant hormonal biosynthesis and signal transduction pathways in mulberry and is highly recommended for use by farmers and the sericulture industries.

## 5. Conclusions

We conclude that the use of differential stubble lengths in mulberry not only promotes early sprouting and increases photosynthetic apparatus of the plant but also improves the contents of endogenous phytohormones and alters the biosynthesis of plant hormones and signal transduction pathways in mulberry plants. Contradictory results were observed among the different stubble lengths, making the selection of the best stubble length a tedious one, but per our projections, 5 cm and 10 cm stubble lengths were selected and recommended for regeneration. Hence, the hypothesis that the use of differential stubble lengths promotes not only the growth of stubbles, but also induces hormones such as GA, IAA, and melatonin, which are known to promote shoot elongation through cell division and differentiation, is accepted. Per the findings of this study, instead of relying on saplings as the only vegetative means of propagation, farmers or the sericulture industries could improve existing mulberry plants for rapid regeneration of leaves by cutting them into stubbles to produce new and succulent leaves for the silkworm. Stubbles have been shown to be a convenient and alternative vegetative method for regenerating, rejuvenating and sprouting leaves and are known to induce various phytohormones that regulate growth and development. This requires critical attention in the futuristic decisions of farmers and, most importantly, the sericulture industries.

## Figures and Tables

**Table 1:** The differential metabolites (DMs) and their expression level (log2_FC).

	log2_FC Values					
Compounds	DXZ-FC-CK-vs-DXZ-FC-0	DXZ-FC-CK-vs-DXZ-FC-5	DXZ-FC-CK-vs-DXZ-FC-10	DXZ-FC-05-vs-DXZ-FC-0	DXZ-FC-10-vs-DXZ-FC-0	DXZ-FC-10-vs-DXZ-FC-5
3-Indolepropionic acid	1.94	2.68	1.96	−0.74		0.72
Jasmonic acid	−0.63	−1.52	−1.28	0.89	0.65	−0.24
Salicylic acid	−2.52	−2.00	−2.46	−0.52	−0.06	0.46
Abscisic acid	−0.76	−0.38	−0.64	−0.38	−0.12	0.26
Gibberellin A1	1.34	1.47	1.73		−0.38	−0.25
Indole-3-acetic acid	1.20	1.57	1.42	−0.37		
Gibberellin A4		0.25	0.39			
2iP Riboside	−0.50	−0.87	−0.78	0.37	0.29	
trans-zeatin-riboside	0.06	−0.42	0.34	0.48	−0.29	−0.77
Trans-zeatin	−0.37	−0.46	−0.20		−0.17	−0.26
Melatonin	0.51		0.28			

Note: DXZ-FC-0 is 0 cm stubble length. DXZ-FC-5 is 5 cm stubble length. DXZ-FC-10 is 10 cm stubble length. Normal (DXZ-FC-CK) is an unstumped mulberry plant designated as CK. Log2_FC is the log transformed values of the fold change (FC) of the differential metabolite expression. Negative and positive values indicate down and upregulated metabolites.

## Data Availability

Data used in this work are described in the article and others are attached as Supplementary Materials.

## Supplementary Materials

The following supporting information can be downloaded at: https://www.mdpi.com/article/10.3390/plants14071126/s1, Table S1: Ratios of hormones determined by biochemical analysis. Table S2: All metabolites expression in leaves from stubble and normal plants. Figure S1: The permutation plot testing of the OPLS-DA score distributions. Figure S2: Volcano plot of differential metabolites (DMs). Figure S3: KEGG pathway annotation with the DMs, etc.
