# Supplementary material for: The Targeted Metabolomic Signatures of Phytohormones in Leaves of Mulberry (Morus alba L.) Are Crucial for Regrowth and Specifically Modulated by the Differential Stubble Lengths
Source: Plants (Basel). 2025 Apr 5;14(7):1126. doi: 10.3390/plants14071126 (PMC11991534; doi:10.3390/plants14071126)
Supplement: Supplementary file 1 [file plants-14-01126-s001.zip › Supplementary Figure.pdf]

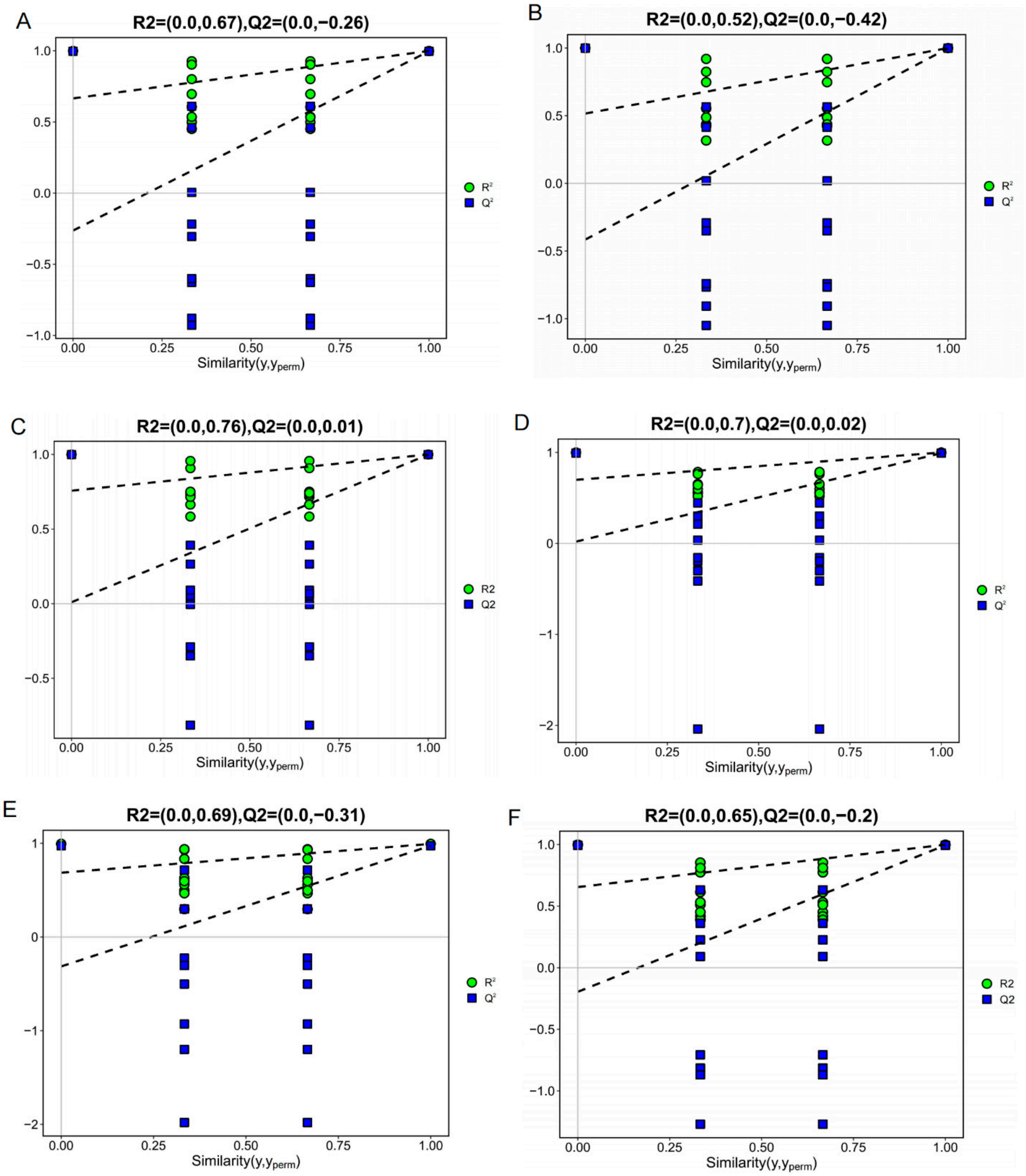

Figure S1. The permutation plot testing of the OPLS-DA score distributions. (A–F) Plots of OPLS-DA permutations in DXZ-FC-CK-vs-DXZ-FC-0, DXZ-FC-CK-vs-DXZ-FC-5, DXZ-FC-CK-vs-DXZ-FC-10, DXZ-FC-5-vs-DXZ-FC-0, DXZ-FC-10-vs-DXZ-FC-0, and DXZ-FC-10-vs-DXZ-FC-5, respectively.

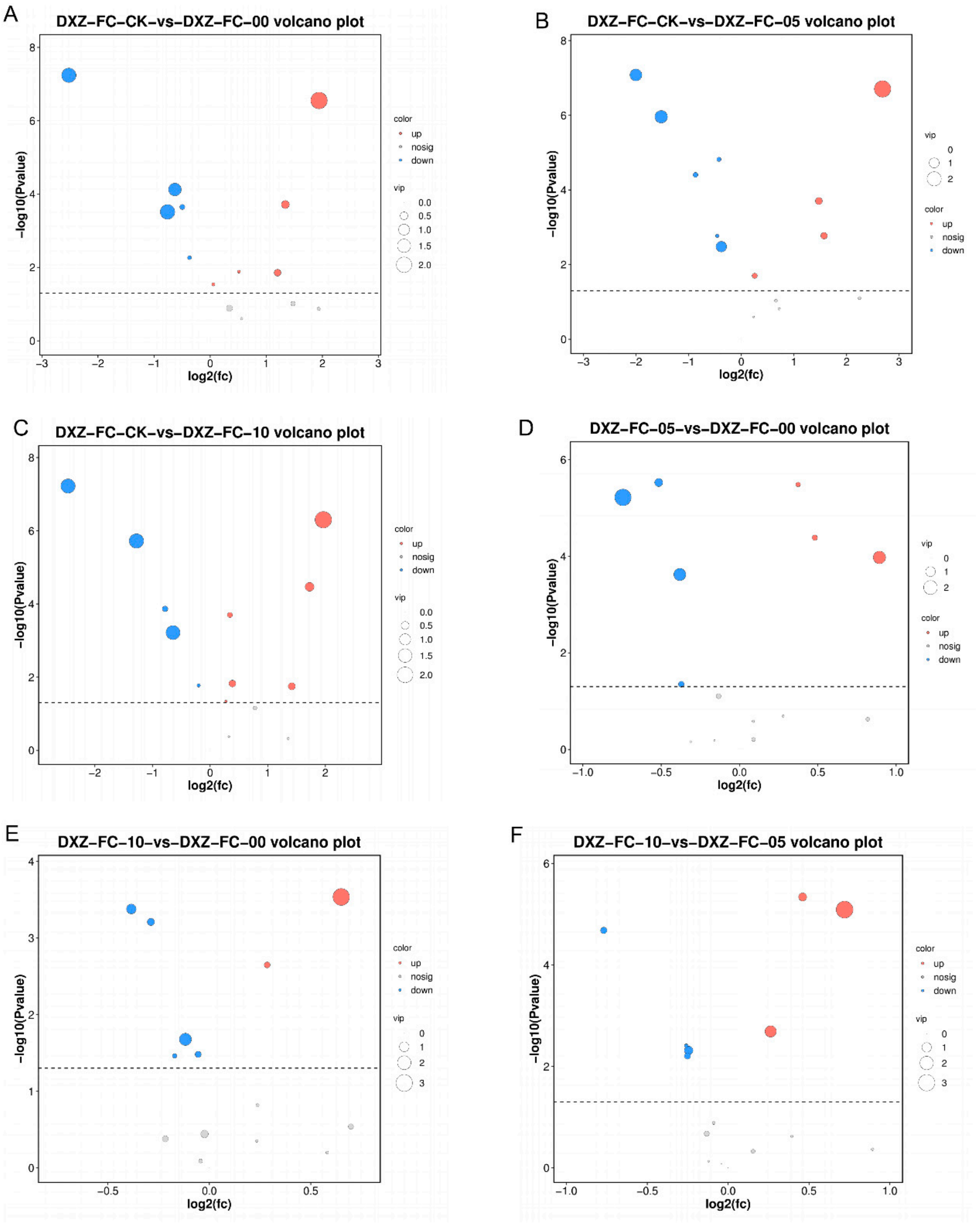

Figure S2. Volcano plot of differential metabolites (DMs). (A–F) Volcano plots of DMs in DXZ-FC-CK-vs-DXZ-FC-0, DXZ-FC-CK-vs-DXZ-FC-5, DXZ-FC-CK-vs-DXZ-FC-10, DXZ-FC-5-vs-DXZ-FC-0, DXZ-FC-10-vs-DXZ-FC-0, and DXZ-FC-10-vs-DXZ-FC-5, respectively. The red color indicates high concentration, and the blue color indicates low concentration. Dashed grey lines indicate the threshold.

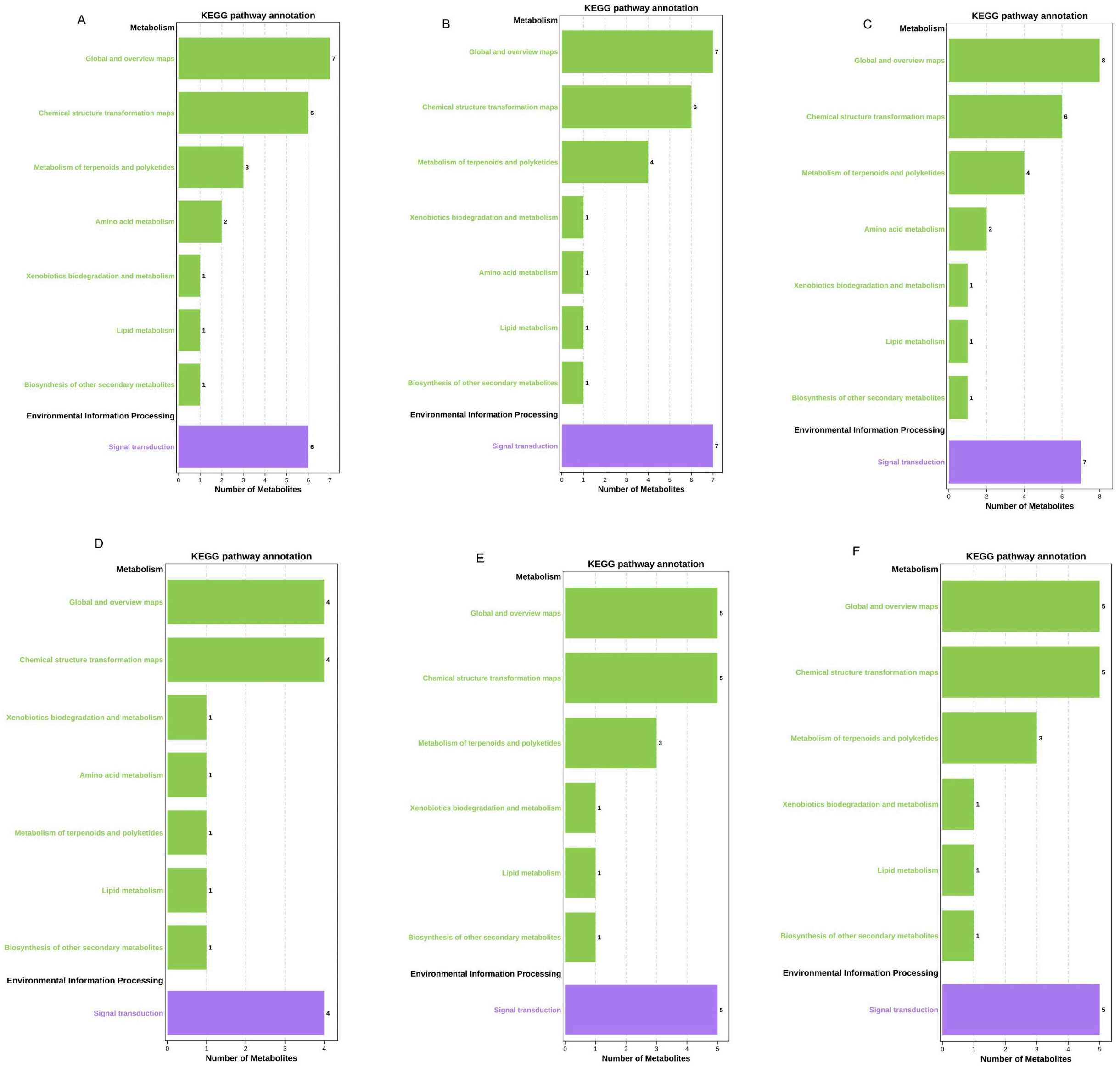

Figure S3. KEGG pathway annotation with the DMs. (A–F) Pathways annotation in DXZ-FC-CK-vs-DXZ-FC-0, DXZ-FC-CK-vs-DXZ-FC-5, DXZ-FC-CK-vs-DXZ-FC-10, DXZ-FC-5-vs-DXZ-FC-0, DXZ-FC-10-vs-DXZ-FC-0, and DXZ-FC-10-vs-DXZ-FC-5, respectively.
